# Prevalence and Contributing Factors of Mandibular Labial Gingival Recession in Patients With Lingual Orthodontic Retainers: A Cross‐Sectional Study

**DOI:** 10.1111/ocr.70045

**Published:** 2025-10-04

**Authors:** Chrysoula Tsiavaki, Effimia Koumpia, Maria Myrsini Pouliou, Leonidas Batas, Ioannis Fragkioudakis, Moschos A. Papadopoulos

**Affiliations:** ^1^ Department of Prosthodontics, Faculty of Dentistry, School of Health Sciences Aristotle University of Thessaloniki Thessaloniki Greece; ^2^ Department of Orthodontics, Faculty of Dentistry, School of Health Sciences Aristotle University of Thessaloniki Thessaloniki Greece; ^3^ School of Dentistry National and Kapodistrian University of Athens Athens Greece; ^4^ Department of Periodontology and Implant Biology, Faculty of Dentistry, School of Health Sciences Aristotle University of Thessaloniki Thessaloniki Greece

**Keywords:** fixed lingual orthodontic retainers, oral habits, orthodontic treatment, periodontics

## Abstract

**Objectives:**

To investigate the prevalence and contributing factors of mandibular labial gingival recession in patients with fixed lingual orthodontic retainers.

**Methods:**

This cross‐sectional study involved 83 post‐orthodontic patients with fixed lingual retainers. Labial gingival recession was assessed on the lower incisors and classified using Cairo's Recession Type system. Clinical parameters were collected, including bleeding on probing, biofilm, brushing habits and oral hygiene behaviours. Logistic regression was used to analyse factors associated with recession.

**Results:**

Labial gingival recession was observed in 27 of 83 patients (32.53%). The mean recession was 0.66 mm. Significant associations were found between gingival recession, age (*p* = 0.008) and nail/pen/pencil biting habits (*p* = 0.021). Brushing technique and frequency also showed a significant association.

**Conclusions:**

Labial gingival recession was present in one‐third of patients with fixed lingual retainers. Age and mechanical habits, such as nail‐biting, were significant predictors. Preventive strategies should target behaviour modification and enhanced oral hygiene education.

## 1 Introduction

Gingival recession is characterised by the apical displacement of the gingival margin from the cemento‐enamel junction (CEJ) and may result in aesthetic concerns, root caries and dentin hypersensitivity [1]. This condition often arises due to periodontal diseases and mechanical trauma, but emerging evidence suggests that orthodontic retainer appliances might also play a significant role [2]. More specifically, fixed lingual retainers, commonly used following orthodontic treatment to maintain occlusal stability, have contributed to labial gingival recession through the buccal movement of the tooth roots and subsequent bone dehiscence [3].

Physiological factors, such as orthodontic movement of teeth to positions outside the labial or lingual alveolar plate, could lead to dehiscence formation [3, 4]. This structural weakness might act as a *locus minoris resistentiae*, predisposing the area to the development of labial gingival recession. Such recession often manifests as deep and narrow lesions akin to ‘Stillman clefts,’ where maintaining oral hygiene becomes challenging, potentially resulting in bacterial or viral infections and the formation of buccal probing pockets reaching the periapical environment of the tooth [5].

Facial soft tissue volume, particularly the gingival thickness, is a key factor in predicting gingival recession during or after orthodontic treatment. A thin gingival phenotype is more vulnerable to recession, especially in the presence of plaque‐induced inflammation or toothbrushing trauma [6, 7, 8]. Therefore, active movement of teeth outside the alveolar bone during orthodontic treatment might be a significant etiological factor for labial gingival recession. During the post‐orthodontic retention phase, wide and deep gingival recessions often occur due to toothbrushing trauma on gingival tissues thinned by tooth malposition and buccal dislocation [9].

There is conflicting evidence regarding the influence of fixed lingual retainers on gingival health, particularly labial gingival recession. Some studies have reported that retainers may contribute to recession due to factors such as plaque retention and mechanical stress [2, 5, 10, 11]. However, others found no significant periodontal differences between retainer types or compared to control groups [12]. This inconsistency underscores the need for further clinical data on the prevalence and contributing factors of labial gingival recession in patients wearing fixed retainers. Additional research concluded that long‐term retention of mandibular incisor alignment was found acceptable for most patients and compatible with periodontal health [13] There is conflicting evidence on the implications of lingually bonded retainers on the prevalence of labial gingival recession and their association with bonded retainers after orthodontic treatment. Therefore, this study aimed to investigate the presence of labial gingival recession in the lower incisor area following orthodontic treatment associated with lingual retainers and the related risk factors.

## 2 Materials and Methods

### 2.1 Study Design

This cross‐sectional study aimed to assess the presence and classification of labial gingival recession (REC) and its association with several factors in patients with a fixed retainer in the mandibular incisor area following orthodontic treatment. Due to the study's cross‐sectional nature and absence of a control group, the present work is limited to observational reporting of recession prevalence and associated factors within a bonded retainer population.

### 2.2 Eligibility Criteria

The study design adhered to established methodologies for periodontal examination and classification of gingival conditions, following the methodology of a previous study [8]. Participants eligible for inclusion in this study were aged between 18 and 30 years, had completed orthodontic treatment more than 1 year before enrollment and were fitted with fixed lingual retainers in the mandibular arch. All participants were required to be fully dentate and exhibit periodontal health or, at most, gingivitis. The selected age range of 18–30 years was chosen to ensure a relatively homogeneous cohort of young adults who had recently undergone orthodontic therapy and were within the early to intermediate stages of post‐treatment retention. This demographic was considered optimal for minimising confounding variables related to age‐associated periodontal changes, such as cumulative tissue loss and most accurately represents individuals likely to retain lingual retainers placed during adolescence.

Exclusion criteria comprised the presence of periodontal pockets ≥ 5 mm, inadequate dental restorations or prostheses, systemic conditions affecting periodontal tissues (e.g., bleeding disorders, diabetes mellitus) or use of medications known to induce gingival overgrowth (e.g., calcium channel blockers, immunosuppressants, anticonvulsants). Individuals with third molars were also excluded to ensure consistent clinical assessments.

Participants were consecutively recruited between September 2023 and May 2024 during routine dental visits at the Department of Periodontology and Implant Biology, Dental School, Aristotle University of Thessaloniki.

### 2.3 Sample Size Calculation

A calculation was performed using consistent data from several studies to determine the sample size for assessing the mean gingival recession in patients with retainers. The mean labial gingival recession was reported as 0.1 mm with a standard deviation of 0.2 mm [12, 13, 14]. Using a one‐sample mean formula with a 95% confidence level (*α* = 0.05) and a margin of error (precision) set at 0.05 mm, the minimum required sample size was calculated as follows:

$$n=\left(\mathrm{EZ}\alpha /2\cdot \sigma \right)2=\left(0.051.96\cdot 0.20\right)2=61.47$$

Rounding up, this yielded a target sample size of 62 participants. To compensate for potential dropouts or unusable data (estimated at 10%), the adjusted sample size was set at 69. A total of 83 participants were ultimately included in the study. This intentional over‐recruitment was performed to increase the statistical power of the analysis and account for potential unforeseen variability in clinical measurements or demographic distribution. Including a larger sample enhances the precision of prevalence estimates and improves the robustness of regression analyses, particularly in an observational study with multiple covariates.

### 2.4 Clinical Examination

All participants underwent a comprehensive full‐mouth clinical examination performed by three calibrated examiners. The examination protocol included:

#### 2.4.1 Gingival Recession (GR) Assessment

GR was measured as the distance between the gingival margin and the cementoenamel junction (CEJ) at the mid‐buccal surface of each lower incisor, focusing on the buccal anterior mandibular area where recession is more prevalent. Gingival recession was classified using the most recent classification system, namely Cairo's Recession Type (RT) classification [14]; RT1: Gingival recession with no loss of interproximal attachment. RT2: Gingival recession associated with loss of interproximal attachment. RT3: Gingival recession with interproximal attachment loss greater than the buccal attachment loss.

#### 2.4.2 Periodontal Health Assessment

Bleeding on Probing (BOP): Recorded as the presence (+) or absence (−) of bleeding, expressed as a percentage, occurring 30 s after a periodontal probe was inserted into the periodontal pocket. Plaque presence or calculus presence with the passing of the probe from the sulcus and visual observation in the anterior mandibular area buccally. Periodontal measurements were obtained using a Hu‐Friedy CP‐15 periodontal probe (Hu‐Friedy, Chicago, IL, USA), which was calibrated in 1 mm increments.

To ensure consistency and accuracy of the measurements, intra‐examiner and inter‐examiner reproducibility were calculated. Calibration sessions were conducted, and the following metrics were used to assess reproducibility: Intra‐examiner Agreement: Calculated with an intraclass correlation coefficient (ICC) for each examiner. The ICC for intra‐examiner agreement was 0.93 (95% CI, 0.89–0.96). Inter‐examiner Agreement: Evaluated by comparing measurements taken by different examiners on the same set of patients. The ICC for the inter‐examiner agreement was 0.91 (95% CI: 0.87–0.95), indicating a high level of consistency among examiners.

### 2.5 Data Collection

A structured questionnaire was administered to obtain detailed information on behavioural and oral hygiene‐related variables. Data collected included: (a) smoking history, encompassing frequency, duration and type of tobacco consumption; (b) oral hygiene practices, specifically toothbrushing technique and frequency; and (c) toothbrush characteristics, such as bristle hardness and type. Participants were also queried regarding the presence of parafunctional habits, including onychophagia (nail biting) and the habitual use of pens or pencils as oral objects.

### 2.6 Statistical Analysis

All statistical analyses were conducted using IBM SPSS Statistics for Windows (Version 29.0). Descriptive statistics were calculated for all variables. The association between the presence of gingival recession (dependent variable) and a range of categorical predictors (e.g., gender, smoking status, brushing technique, interdental cleaning habits, toothbrush type, oral hygiene frequency, harmful oral habits, presence of bleeding on probing [BOP], frenulum and keratinized tissue) was initially assessed using Pearson's chi‐square test of independence. Continuity corrections and Fisher's exact test were applied for 2 × 2 tables when appropriate. Contingency coefficients were used as measures of effect size for nominal variables. Variables with statistically significant associations (*p* < 0.05) in the bivariate analyses were then included in a multivariate logistic regression model to identify independent predictors of gingival recession. The logistic regression was performed using the enter method, with odds ratios (ORs) and 95% confidence intervals (CIs) calculated. Goodness‐of‐fit was assessed with the Hosmer–Lemeshow test, and model explanatory power was evaluated via Cox & Snell and Nagelkerke *R* ^2^. Statistical significance was set at *p* < 0.05. Before performing logistic regression, multicollinearity diagnostics were conducted using linear regression to calculate variance inflation factors (VIFs). All predictors had VIF values below two and tolerances above 0.5, indicating no evidence of problematic multicollinearity among independent variables.

## 3 Results

### 3.1 Demographics of Participants

A total of 126 patients were screened for eligibility between September 2023 and May 2024. Of these, 3 patients declined participation and 40 were excluded: 7 due to periodontal pockets ≥ 5 mm, 12 due to systemic conditions or medications affecting periodontal tissues, 9 due to the presence of third molars and 12 because they were outside the age range of 18–30 years. The study included 83 participants with the following demographic characteristics: the mean age was 25.67 years, with 34 males and 49 females. Among the participants, 20 were smokers and 63 were non‐smokers. The mean duration of the retention phase (time since placement of the fixed lingual retainer) was 7.92 ± 5.63 years (95% CI: 6.66–9.18; range: 1–22 years; median: 8 years).

### 3.2 Prevalence of Labial Gingival Recession

Of the 83 participants, 27 had a labial gingival recession, resulting in a presence rate of 32.53%. This indicates that one‐third of participants were affected by labial gingival recession, highlighting its common occurrence in this population. Detailed prevalence data are provided in Table 1. Among the 27 patients with labial gingival recession, the condition was limited to one lower incisor in 14 cases (51.8%) and involved two adjacent incisors in 9 cases (33.3%). In only four patients, generalised recession involving more than two teeth was observed (14.8%). The most frequently affected teeth were the central incisors.

**TABLE 1:** Association between demographic, behavioural and clinical variables and the presence of gingival recession: Chi‐square test of independence with corresponding degrees of freedom (df), *p*‐values and contingency coefficients (CC).

Variable	*χ* ^2^ (df)	*p*	Contingency coefficient (CC)
Gender	0.353 (2)	0.838	0.066
Smoking	1.771 (1)	0.183	0.147
Brushing technique	12.613 (3)	**0.006**	0.369
Brushing frequency	17.584 (3)	**< 0.001**	0.424
Interdental cleaning	0.002 (1)	0.965	0.005
Toothbrush hardness	0.147 (1)	0.702	0.043
Parafunctional habits	5.290 (1)	**0.021**	0.249
Bleeding on probing	4.214 (1)	**0.040**	0.224
Frenulum attachment	2.229 (1)	0.135	0.165

*Note:* Bold *p*‐values indicate statistical significance at *p* < 0.05. The contingency coefficient (CC) indicates the strength of association between each variable and gingival recession (values closer to 1 indicate stronger association).

### 3.3 Categories of Gingival Recession

The distribution of labial gingival recession among the participants was as follows: 21 cases were classified as RT 1 and 6 as RT 2. This classification helps understand the severity and extent of the recession observed by the participants. RT 1 refers to recession with no interproximal attachment loss, whereas RT 2 indicates recession with some interproximal attachment loss.

### 3.4 Summary of Gingival Recession Measurements

The mean labial gingival recession in the mandibular incisor area was 0.66 mm, with a standard deviation of 1.17 mm. The minimum recorded recession was 0 mm, and the maximum was 5 mm. These measurements provide a quantitative assessment of the severity of gingival recession in the study population. The wide range of recession values indicates variability in the severity of the condition among the participants.

### 3.5 Factors Associated With Gingival Recession

Crosstabulations using Pearson's chi‐square test and contingency coefficients were performed to evaluate associations between gingival recession (GR) and various demographic, behavioural and clinical variables. A summary of the statistical results is presented in Table 1.

Brushing technique was significantly associated with GR (*χ* ^2^(3) = 12.613, *p* = 0.006), with individuals who used a circular brushing motion exhibiting the highest prevalence of recession (64.5%). Brushing frequency was also strongly related to GR (*χ* ^2^(3) = 17.584, *p* < 0.001), with participants brushing less than twice daily showing a markedly higher incidence of GR. These behavioural factors demonstrated moderate‐to‐strong associations, as reflected by contingency coefficients (CC = 0.369 and CC = 0.424, respectively).

Parafunctional oral habits (e.g., nail biting or bruxism) were significantly more frequent among participants with GR (*χ* ^2^(1) = 5.290, *p* = 0.021; CC = 0.249), suggesting a role for mechanical trauma in marginal tissue damage. Bleeding on probing (BOP), an indicator of inflammation, was also significantly associated with GR (*χ* ^2^(1) = 4.214, *p* = 0.040; CC = 0.224).

The strongest association was observed between GR and the absence of keratinized gingiva (*χ* ^2^(1) = 24.600, *p* < 0.001), with 51.6% of participants with GR lacking keratinized tissue, compared to only 4.1% in those without recession. The high contingency coefficient (CC = 0.485) supports a robust relationship, underscoring the clinical relevance of keratinized tissue in maintaining gingival margin stability.

Other variables, including gender, smoking status, interdental cleaning, toothbrush hardness and frenulum position, did not show statistically significant associations with GR (all *p* > 0.05). These findings suggest that specific behavioural and clinical factors, especially oral hygiene practices and soft tissue characteristics, may have a greater influence on GR than demographic variables alone.

To further identify independent predictors, a binary logistic regression was performed including age, time since orthodontic treatment and variables significantly associated with GR in the bivariate analysis. The model was statistically significant (*χ* ^2^(17) = 65.205, *p* < 0.001) and demonstrated excellent predictive power (Nagelkerke *R* ^2^ = 0.761). Model calibration was adequate, as indicated by a non‐significant Hosmer–Lemeshow test (*p* = 0.129), and the model correctly classified 92.4% of cases.

In the multivariate analysis, age emerged as a significant positive predictor of GR (OR = 1.233, 95% CI: 1.057–1.438, *p* = 0.008), indicating a 23.3% increase in odds of recession for each additional year of age. Conversely, the presence of keratinized gingiva significantly reduced the likelihood of recession (OR = 0.006, 95% CI: 0.000–0.231, *p* = 0.006). Other variables lost significance in the adjusted model, possibly due to multicollinearity or confounding effects.

## 4 Discussion

Gingival recession, a prevalent dental condition, refers to the apical migration of the gingival margin, exposing the roots of the teeth [1]. This exposure can lead to increased tooth sensitivity, a higher risk of dental caries and aesthetic concerns [2]. Understanding the prevalence, demographic factors and the impact of fixed orthodontic retainers on labial gingival recession is crucial for developing effective prevention and management strategies.

This study found a 32.53% prevalence of labial gingival recession among participants, which is consistent with similar studies reporting comparable rates, depending on the population and criteria used. In a 2010 study, a prevalence of up to 59.1% was reported in patients with long‐term use of fixed retainers, indicating that the duration of retainer use significantly impacts the development of gingival recession [5]. The findings of the current study are consistent with those of Levin et al., who found that teeth with fixed retainers had significantly greater mean gingival recession (0.09 ± 0.2 mm) compared to those without (0.01 ± 0.1 mm) [13]. This suggests that fixed retainers contribute to gingival recession by creating areas prone to plaque accumulation and challenging oral hygiene. Conversely, in a recent systematic review, 73.3% of young orthodontically treated patients with fixed steel retainers exhibited healthy gingival conditions after 1 year, which is comparable to the rate in the control group (88.2%). It was concluded that while some studies showed minimal gingival recession associated with fixed retainers, the evidence is not robust enough to establish a clear association due to study design variations, retainer use duration and oral hygiene practices [12]. Furthermore, it has been suggested that the distance between the gingival margin and the retainer (mean distance of 5 mm) could be responsible for the inconspicuous periodontal parameters observed in the test group [2, 12].

In the current study, age was a significant contributor to labial gingival recession in patients with fixed lingual orthodontic retainers. A significant positive association was found between age and labial gingival recession (*p* = 0.008); older age was associated with an increased likelihood of recession. This suggests that individuals are more prone to developing labial gingival recession as they age, possibly due to cumulative exposure to risk factors and natural aging processes affecting the gingival tissues. Nevertheless, age has been previously identified as a significant factor in the development of labial gingival recession [1]. Studies indicate that 88% of people over the age of 65 experience gingival recession on one or more teeth, suggesting a direct correlation between age and the likelihood of gingival recession [15]. This increase can be attributed to long‐term exposure to periodontal pathogens, mechanical wear and a potential decline in oral hygiene over time. Older adults also tend to retain their teeth longer, further increasing the risk of recession due to prolonged exposure to various risk factors [16]. Additionally, gingival thickness, which tends to decrease with age, is crucial in gingival recession. Thicker gingiva provides better resistance against mechanical and bacterial challenges, while thinner gingiva is more susceptible to recession [17].

Nail or pen/pencil biting habits showed a significant positive association with labial gingival recession (*p* = 0.021), increasing the likelihood of recession. Aggressive brushing techniques can lead to mechanical trauma, causing the gingival margin to recede. It is recommended to use soft‐bristled toothbrushes and gentle brushing techniques to prevent such damage [6]. It has been proposed that habits like nail biting exacerbate labial gingival recession, especially in individuals with fixed orthodontic retainers [3]. The combination of mechanical trauma from habits and plaque retention around retainers creates an environment conducive to gingival recession. Moreover, habits such as bruxism (teeth grinding) and improper use of toothpicks or dental floss can lead to continuous mechanical stress on the gingival tissues, resulting in gingival recession. These habits contribute to recession by causing repeated trauma to the gingival margins, which, over time, leads to tissue breakdown and recession [9, 15]. In a pilot study conducted by Nguyen‐Hieu et al., it was found that young individuals who engaged in nail‐biting or used improper brushing techniques had a higher incidence of gingival recession [18]. This underscores the importance of educating patients about proper oral hygiene practices and the risks associated with harmful habits [18].

The use of brushing methods other than the Bass technique was marginally associated with an increased risk of labial gingival recession (*p* = 0.06). Proper brushing techniques should be emphasised to minimise mechanical trauma to the gingiva [19].

Several other factors, including smoking, interdental cleaning habits (e.g., dental floss or interdental brushes), brushing frequency, presence of biofilm/calculus, BOP, frenulum attachment, keratinized gingiva, gender and type of toothbrush used, were not significantly associated with labial gingival recession. In this study, out of 83 patients, 20 were identified as smokers, with the majority (17 out of 20) classified as light smokers, consuming fewer than ten cigarettes per day. This demographic detail is critical when discussing the influence of smoking on gingival recession. While smoking is a well‐documented risk factor for periodontal diseases, the minimal daily intake among our subjects may contribute to a reduced impact on gingival health. Previous research has highlighted a dose–response relationship, where the severity of periodontal disease escalates with increased cigarette consumption [20]. Additionally, lighter smoking has been associated with less severe periodontal outcomes compared to heavier smoking [21, 22]. Therefore, the low intensity of smoking among our patients suggests that the observed gingival recession might be minimally influenced by smoking. This is consistent with findings that indicate lower levels of smoking are less likely to cause significant periodontal damage [23]. These insights highlight the importance of considering smoking intensity in assessing its impact on periodontal health. The lack of significant association with smoking contradicts some previous findings, possibly due to differences in study populations, methods or uncontrolled confounding factors [6, 13]. In this study, traditionally considered potential risk factors were not associated with gingival recession, which may indicate that their impact is less pronounced or more complex than previously thought.

An emerging orthodontic complication that merits consideration in the context of our findings is *wire syndrome* (WS). WS describes atypical and often progressive tooth displacements occurring despite the presence of an intact, bonded orthodontic retainer, without fracture or debonding [24]. These movements are typically classified as torque‐related asymmetries, such as the ‘X‐effect’ between adjacent incisors or ‘Twist‐effect’ involving contralateral canines. WS has been associated with deleterious periodontal outcomes, including gingival thinning, Cairo RT1/RT2 recessions, fenestrations and even complete root exposure in advanced cases. Kučera and Marek reported an unexpected complication prevalence of about 1.1% among 3500 patients with mandibular fixed retainers. Most complications manifested as canine tipping (twist effect) or incisor torque differences (X effect), often caught approximately 4 years into retention. Facial divergence (higher mandibular plane angle) and a more ventrally positioned incisor pretreatment were significant predictors, while wire type and treatment duration were not [22, 24]. Given that our study documented labial recession frequently affecting mandibular incisors in patients with intact fixed retainers, it is plausible that undetected cases of early or moderate WS may have contributed to these findings. This highlights the need for clinicians to differentiate WS from classic orthodontic relapse through close post‐treatment monitoring, including torque assessment and photographic comparisons over time.

The findings of this study highlight the multifactorial nature of gingival recession, which involves both age‐related physiological changes and mechanical factors related to personal habits. Addressing these factors through targeted prevention and patient education can significantly reduce the risk and progression of labial gingival recession. Clinicians should emphasise the importance of proper oral hygiene techniques and regular dental visits to monitor and manage the early signs of labial gingival recession. Additionally, the design and placement of fixed retainers should minimise the risk of plaque retention and promote periodontal health during and after orthodontic treatment.

A significant limitation of this study is the absence of a control group consisting of patients without fixed lingual retainers. As a result, while the prevalence of labial gingival recession in this population is notable, no causal relationship between retainers and recession can be established. Future studies should include a matched control group to facilitate comparative analysis and strengthen inferences. An additional limitation of the study is that gingival recession was evaluated only on the labial surfaces of the mandibular incisors. Lingual surfaces were not assessed, which prevents a direct comparison between facial and lingual recession patterns. While the labial surface is more clinically relevant due to visibility and sensitivity, future research should include both aspects for a more comprehensive periodontal evaluation. Another limitation of the present study is the lack of assessment of gingival phenotype. Although gingival thickness is well established as a critical factor influencing susceptibility to gingival recession, it was not evaluated in our cohort. This omission was primarily methodological: reproducible parameters such as Cairo's classification, bleeding on probing and the presence of keratinized tissue were prioritised because they could be standardised across examiners with high calibration reliability. In contrast, gingival phenotype assessment would have required additional invasive or specialised methods (e.g., transgingival probing or ultrasonography) that were not feasible to apply consistently in the present clinical setting. This study's cross‐sectional design limits the ability to establish causality. A significant limitation of this study is the lack of periodontal data prior to orthodontic treatment. Although the inclusion criteria required that all participants be currently periodontally healthy or have gingivitis at the time of examination—ensuring a homogeneous clinical status during data collection—no information was available regarding their periodontal condition before or during orthodontic therapy. As a result, it remains uncertain whether the observed labial gingival recession developed after treatment or was already present prior to treatment. This limitation is inherent to the cross‐sectional design, which captures outcomes at a single time point without temporal comparisons. Future longitudinal studies are essential to clarify the causal relationship between fixed retainers, changes in tooth inclination and the development of labial gingival recession. Such research should include standardised periodontal assessments conducted before, during and after orthodontic treatment to track changes in soft tissue over time. Additionally, this study lacked information on the severity of initial malocclusions, particularly mandibular anterior crowding, which may play a crucial role in recession development due to associated alveolar bone dehiscence or unfavourable tooth movements. The inability to assess this potential confounder limits the interpretation of our findings. Furthermore, most participants in the present study had completed orthodontic treatment within a short to intermediate timeframe. Longer retention periods may result in more pronounced or cumulative soft tissue alterations. Therefore, future studies should examine gingival outcomes across varied retention durations and among larger, more diverse populations to better understand long‐term periodontal implications.

## 5 Conclusion

In this study, almost one‐third of the participants presented labial gingival recession, with more than one‐third of patients affected. Age and nail/pen/pencil biting habits were significantly associated with labial gingival recession in this sample of patients with fixed lingual orthodontic retainers. However, due to the cross‐sectional design and absence of a control group, these associations should be interpreted with caution. Preventive strategies should focus on proper oral hygiene education and regular periodontal maintenance, especially for older adults with fixed lingual retainers.

Future research should elucidate the underlying biological mechanisms and the interplay between various risk factors contributing to labial gingival recession. Longitudinal studies with larger and more diverse populations are needed to confirm these findings and develop comprehensive prevention and treatment strategies for gingival recession in patients with fixed lingual orthodontic retainers.

## Disclosure

The authors did not use generative AI or AI‐assisted technologies in the preparation of this manuscript.

## Ethics Statement

This study was conducted in accordance with the ethical standards of the Ethics Committee of the Faculty of Dentistry, Aristotle University of Thessaloniki (Protocol number: 38/16‐05‐2016) and in compliance with the 1975 Declaration of Helsinki and its subsequent amendments.

## Consent

All participants were informed of the study's purpose and procedures, and written informed consent was obtained from each participant before their inclusion.

## Conflicts of Interest

The authors declare no conflicts of interest.

## Data Availability

The data that support the findings of this study are available from the corresponding author upon reasonable request. The dataset includes anonymized clinical and questionnaire‐based variables used in the statistical analysis.
